# Association of intraoperative hypotension and acute kidney injury in noncardiac surgery patients: a post hoc secondary analysis of the EU HYPROTECT registry

**DOI:** 10.1007/s10877-025-01329-4

**Published:** 2025-07-17

**Authors:** Julian Runge, Carla D. Grundmann, Carolina Mucha, Robin Denz, Karim Kouz, Manuel Ignacio Monge García, Elisabetta Cerutti, Luciano Frassanito, Michael Sander, Simon J. Davies, Abele Donati, Javier Ripolles-Melchor, Daniel García-Lopez, Benjamin Vojnar, Etienne Gayat, Eric Nol, Tim van den Boom, Peter Bramlage, Bernd Saugel, Thomas W. L. Scheeren, Ulrich H. Frey

**Affiliations:** 1 Department of Anaesthesiology, Operative Intensive Care Medicine, Pain and Palliative Medicine, Marien Hospital Herne, Ruhr-University Bochum, Herne, Germany; 2 Department of Medical Informatics, Biometry and Epidemiology, Ruhr- University Bochum, Bochum, Germany; 3 Department of Anesthesiology, Center of Anesthesiology and Intensive Care Medicine, University Medical Center Hamburg-Eppendorf, Hamburg, Germany; 4 Unidad de Cuidados Intensivos, Hospital Universitario de Puerto Real, Puerto Real, Spain; 5 Department of Anesthesia, Transplant and Surgical Intensive Care, Azienda Ospedaliero Universitaria Delle Marche, Ancona, Italy; 6 Department of Emergency, Intensive Care Medicine and Anesthesia, IRCCS Fondazione Policlinico Universitario Agostino Gemelli, Rome, Italy; 7 Department of Anaesthesiology, Intensive Care Medicine and Pain Medicine, University Hospital Giessen, Justus-Liebig University Giessen, Giessen, Germany; 8 York and Scarborough Teaching Hospitals NHS Foundation Trust, York, UK; 9 Centre for Health and Population Sciences, Hull York Medical School, York, UK; 10 Department of Biomedical Sciences and Public Health, Universita Politecnica Delle Marche, Ancona, Italy; 11 Department of Anesthesia, Hospital Universitario Infanta Leonor, Madrid, Spain; 12 Universidad Complutense de Madrid, Madrid, Spain; 13 Department of Anaesthesiology and Reanimation, University Hospital Marques de Valdecilla, Santander, Spain; 14 Department of Anaesthesiology and Intensive Care Medicine, University Hospital Marburg, Marburg, Germany; 15 Université Paris Cité, INSERM, Paris, France; 16 Department of Anesthesia and Critical Care Medicine, Hopital Lariboisiere, Paris, France; 17 Department of Anesthesiology and Intensive Care, Hopital de Hautepierre, Les Hôpitaux Universitaires de Strasbourg, Strasbourg, France; 18 Edwards Lifesciences, Nyon, Switzerland; 19 Institute for Pharmacology and Preventive Medicine, Cloppenburg, Germany; 20 BD Advanced Patient Monitoring, Heidelberg, Germany; 21 Department of Anesthesiology, University Medical Center Groningen, University of Groningen, Groningen, The Netherlands

**Keywords:** Blood pressure, Cardiovascular dynamics, Haemodynamic monitoring, Postoperative complications, Renal failure

## Abstract

**Purpose:**

Previous cohort studies suggest that intraoperative hypotension is associated with acute kidney injury (AKI) in noncardiac surgical patients. We sought to ascertain that intraoperative hypotension is independently associated with AKI within the first 3 days after surgery in a contemporary cohort of noncardiac surgery patients in whom clinicians strove to avoid profound intraoperative hypotension.

**Methods:**

This was a post hoc secondary analysis of the multicentre EU HYPROTECT registry, which includes patients undergoing major noncardiac surgery who underwent predictive blood pressure monitoring. The primary outcome of this secondary analysis was AKI within the first 3 days after surgery. To quantify the duration and severity of intraoperative hypotension we calculated the area under a mean arterial pressure (MAP) of 65 mmHg. We used logistic regression analysis to identify factors independently associated with AKI.

**Results:**

We analysed 697 patients. 62 of these 697 patients (9%) developed AKI within the first 3 days after surgery. In multivariable binary logistic regression analysis adjusted for confounding variables, the area under a MAP of 65 mmHg was independently associated with AKI within the first 3 days after surgery (OR 1.03 [95% CI 1.01–1.05] per 10 mmHg*min; *P* < 0.001).

**Conclusion:**

Our secondary analysis of the EU HYPROTECT registry shows that, in a contemporary population of noncardiac surgery patients in whom clinicians strove to avoid profound intraoperative hypotension, intraoperative hypotension is independently associated with AKI within the first 3 days after surgery.

## Introduction

Numerous cohort studies have consistently suggested that intraoperative hypotension is associated with acute kidney injury (AKI) in patients undergoing noncardiac surgery [1, 2, 3, 4, 5, 6, 7, 8, 9, 10]. The risk of developing AKI increases with increasing severity and duration of intraoperative hypotension [1, 2, 3, 4, 5, 6, 7, 8, 9, 10]. Baseline risk factors– such as age and comorbidities– are more strongly associated with AKI than intraoperative hypotension [7, 8]– but are not modifiable. In contrast, intraoperative blood pressure is a modifiable factor. As the population harm threshold for AKI appears to be a mean arterial pressure (MAP) of around 65 mmHg [8, 11], it is recommended to maintain intraoperative MAP above this level [12, 13].

The results of previous cohort studies [8, 9, 11] and recent recommendations [12, 13] may have influenced how clinicians manage blood pressure during surgery. Consequently, patients undergoing surgery today may experience less intraoperative hypotension [14] than patients who underwent surgery over a decade ago and were subsequently included in retrospective cohort studies [14]. There is a paucity of evidence regarding the association between intraoperative hypotension and AKI in contemporary patient populations.

We thus aimed to assess if intraoperative hypotension is independently associated with AKI within the first 3 days after surgery in a contemporary cohort of noncardiac surgery patients in whom clinicians strove to avoid profound intraoperative hypotension. We used data from the multicentre EU HYPROTECT registry [15] that includes major noncardiac surgery patients who had predictive blood pressure monitoring and in whom profound hypotension was rare [16]. In this cohort, the median time-weighted average MAP < 65 mmHg was 0.03 mmHg, and approximately 40% of patients experienced no episode of MAP < 65 mmHg lasting at least one minute [16].

## Methods

### Study design and setting

This is a post hoc secondary, retrospective cohort analysis of the prospective multicentre EU HYPROTECT registry [15, 16]. The registry was approved by the ethics committees at each site and registered at ClinicalTrials.gov (NCT04972266) on July 22, 2021. Patients gave written informed consent to participate in the registry (unless the local ethics committee waived the need for informed consent).

We previously published the details of the study protocol and methods [15], the primary results [16], and another secondary analysis [17]. Briefly, EU HYPROTECT is a European, multicentre registry including adults who had Acumen Hypotension Prediction Index software (HPI-software) monitoring (Edwards Lifesciences; Irvine, CA, USA) during elective major noncardiac surgery in 12 medical centres in 5 European countries (France, Germany, Italy, Spain, and the United Kingdom) [15, 16].

### Participants

**Fig. 1:**
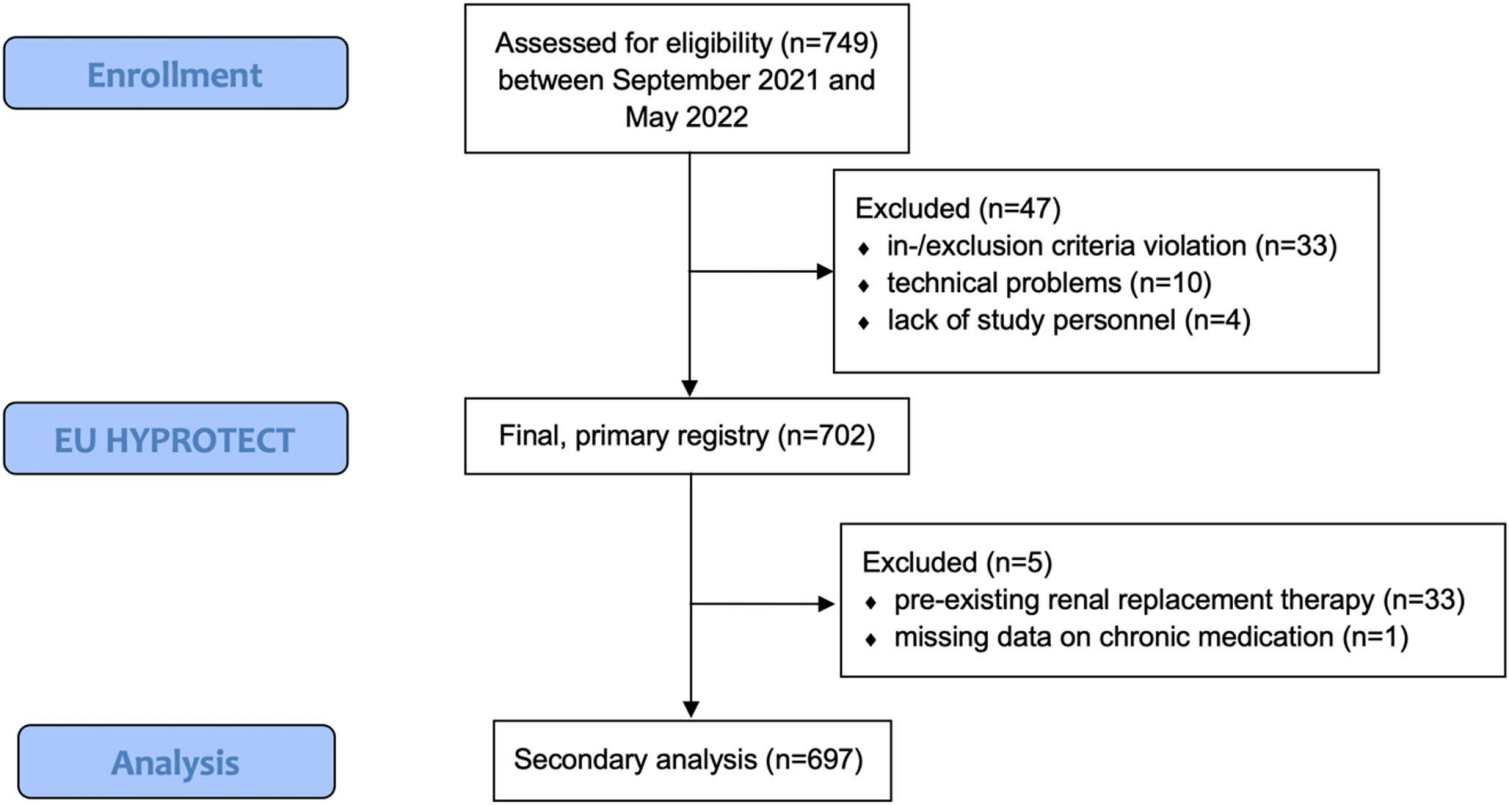
Study Flow Diagram. Of the 749 patients initially enrolled in the EU HYPROTECT registry, 47 were excluded due to protocol violations, technical issues, or unavailable study personnel, resulting in 702 eligible patients. For this secondary analysis, an additional 5 patients were excluded (4 with pre-existing renal replacement therapy, 1 with missing antihypertensive medication data), yielding a final cohort of 697 patients for this secondary analysis

Between September 2021 and May 2022, 749 patients were included in the EU HYPROTECT registry. After the exclusion of 47 patients due to inclusion or exclusion criteria violation, technical problems or lack of study personnel to initiate monitoring, the registry finally includes 702 patients [15] (Fig. 1). Registry patients had elective major noncardiac surgery under general anaesthesia that was expected to last at least 120 min. All patients had intra-arterial blood pressure and HPI-software monitoring. Patients were excluded from the original registry if they had emergency surgery, nephrectomy, liver or kidney transplantation; had atrial fibrillation and/or sepsis (according to current Sepsis-3 definition); were designated American Society of Anesthesiologists (ASA) physical status class V or VI; were not able to understand the nature, significance, and scope of the investigation; were pregnant; did not sign informed consent; and participated in interventional trials. In this secondary analysis, we excluded a further five of the 702 patients: Four because they required renal replacement therapy prior to being included in the study, and one because data on the long-term use of antihypertensive medication was missing. A total of 697 patients were thus included.

### Exposure

Blood pressure was measured with a radial arterial catheter and data extracted from the HemoSphere monitoring platform (Edwards Lifesciences). Data collection was initiated at surgical start and discontinued at the end of surgery. Centres that routinely use this technology participated, and treatment followed local clinical practice without a standardized protocol. To quantify the duration and severity of intraoperative hypotension, we calculated the area under a MAP of 65 mmHg (unit: mmHg*min) and the time-weighted average MAP < 65 mmHg (unit: mmHg). The time-weighted average MAP < 65 mmHg is the area under a MAP of 65 mmHg divided by the total monitoring time.

### Primary endpoint and outcome

The primary outcome of this secondary analysis was AKI within the first 3 days after surgery. We defined AKI based on the “Kidney Disease: Improving Global Outcomes Clinical Practice Guideline for Acute Kidney Injury” [18, 19] as (a) an increase in serum creatinine concentration of ≥ 0.3 mg/dL within any 48 h within the first 3 postoperative days, (b) an increase in serum creatinine of ≥ 50% from baseline within the first 3 postoperative days, or (c) the need for renal replacement therapy within the first 3 postoperative days. We considered serum creatinine values when measured per routine care before surgery (baseline) and on postoperative days 1 to 3. Only patients with at least one preoperative and one postoperative (day 1–3) serum creatinine value were included in this secondary analysis. We did not consider urine output to diagnose AKI in accordance with current recommendations [18, 19] because urine output is usually not reliably recorded after surgery.

### Statistical methods

Demographical and intraoperative data are described separately for patients with and without AKI. Categorical variables are presented as absolute numbers (percentages). We evaluated continuous variables for normal distribution using histograms and quantile-quantile plots, and presented normally distributed continuous variables as mean (standard deviation (SD)) and non-normally distributed continuous variables as median (25th percentile, 75th % percentile).

We used logistic regression analysis to identify factors independently associated with AKI. We first assessed crude (unadjusted) odds ratios (OR) in univariable logistic regression analysis. The initial set of potential confounders was selected based on clinical relevance and prior literature, and included demographic variables (e.g., age, sex), comorbidities (e.g., arterial hypertension, diabetes, chronic kidney disease), baseline renal function, type and duration of surgery, and intraoperative MAP characteristics. We then performed multivariable logistic regression analysis adjusted for the confounders age, body mass index, ASA physical status class, arterial hypertension, pre-existing chronic kidney injury, diabetes mellitus, and total duration of surgery to assess the effect of the area under a MAP of 65 mmHg on AKI within the first 3 days after surgery. These confounders were selected based on univariable analysis (factors with a P-value of less than 0.05 in univariate analysis) and published recommendations [20]. Data are presented as OR (95% confidence intervals (95% CI)) and associated P-values. To facilitate comprehension of the area under a MAP of 65 mmHg values (initially expressed in mmHg*min), odds ratios are shown for increments of 10 mmHg*min. Therefore, the reported odds ratio represents the area under a MAP of 65 mmHg over 10 min, meaning that each reported OR represents the change in odds of AKI associated with an additional cumulative exposure of 10 mmHg*min below the MAP threshold of 65 mmHg. This was deemed an appropriate allocation given the mean duration of surgery of about 200 min.

All tests were two-sided, and statistical significance was considered with a P-value < 0.05. The statistical analyses were performed using SPSS 28.0 (IBM, Armonk, NY, USA) and R 4.2.2 GUI 1.79 Big Sur ARM build (R basis for statistical calculation; Vienna University of Economics and Business, Vienna, Austria) using the packages “tableone”, “tidyverse”, “rio”, “gtsummary”, “marginaleffects”, “reshape2”, “labelled”, “DescTool”, “pander”, “gplot2”, “haven”, and “dplyr”.

## Results

749 patients were screened in the EU HYPROTECT registry. After exclusion 47 patients in the primary registry and exclusion of 5 patients for this analysis, we finally included a total of 697 patients in this secondary analysis.

Mean age was 62.7 ± 12.9 years, and 51.5% of the patients were male. During surgery with a median duration of 199 (141, 275) minutes, the median area under a MAP of 65 mmHg was 6.1 (0.0, 42.2) mmHg*min, and the median time-weighted average MAP < 65 mmHg was 0.03 (0.00, 0.20) mmHg.

62 of the 697 patients (9%) developed AKI within the first 3 days after surgery. Table 1 shows demographic and clinical data separately for patients with and without AKI within the first 3 days after surgery. Compared to patients who did not develop AKI within the first 3 days, patients who did were older (67.7 versus 62.2 years), were more frequently designated ASA physical status class III and IV (66 vs. 40%), more often had arterial hypertension (64.5 versus 50.2%) and pre-existing chronic kidney injury (19.4 versus 4.1%), and had surgery for a longer time (210 versus 196 min). Patients who developed AKI within the first 3 days also had more intraoperative hypotension than patients who did not (area under a MAP of 65 mmHg: 25.37 versus 5.44 mmHg*min; and time-weighted average MAP < 65 mmHg: 0.14 versus 0.03 mmHg) (Table 2).

.

**Table 1:** Demographical and clinical data

Variable	No-AKI (*n* = 635)	3-day-AKI (*n* = 62)
Age (yr)	62.2 (13.1)	67.7 (11.7)
Male sex, n	321 (50.6)	38 (61.3)
Weight (kg)	75 (65, 89)	78 (70, 98)
Height (cm)	169 (163, 177)	170 (164, 178)
Body mass index (kg/m^2^)	26.04 (23.41, 29.37)	26.99 (24.25, 32.58)
Body surface area (m^2^)	1.86 (1.71, 2.03)	1.87 (1.76, 2.07)
ASA physical status class, n I	26 (4.1)	1 (1.6)
II	354 (55.7)	20 (32.3)
III	253 (39.8)	39 (62.9)
IV	2 (0.3)	2 (3.2)
Arterial hypertension, n	319 (50.2)	40 (64.5)
Diabetes mellitus, n	89 (14.0)	14 (22.6)
Chronic obstructive pulmonary disease, n	65 (10.2)	6 (9.7)
Congestive heart failure, n	16 (2.5)	1 (1.6)
Coronary artery disease, n	45 (7.1)	6 (9.7)
Prior stroke/transient ischemic attack, n	22 (3.5)	3 (4.8)
Chronic kidney injury, n	26 (4.1)	12 (19.4)

Categorical data are presented as absolute number (percentage), continuous data are presented as mean (standard deviation) in case of normal distribution, otherwise, as median (25th percentile, 75th percentile)

AKI, acute kidney injury; ASA, American Society of Anesthesiologists

**Table 2:** Intraoperative data

Variable	No-AKI (*n* = 635)	3-day-AKI (*n* = 62)
Total procedure time (min)	196 (140, 272)	210 (174, 315)
MAP (mmHg)	99 (90, 108)	101 (89, 113)
Time-weighted average MAP < 65 mmHg (mmHg)	0.03 (0.00, 0.18)	0.14 (0.00, 0.46)
Area under a MAP of 65 mmHg (mmHg*min)	5.44 (0.00, 35.62)	25.37 (0.97, 144.05)
Relative duration (% of procedure time) with a MAP < 65 mmHg (%)	0.88 (0.00, 4.13)	3.83 (0.00, 7.95)
Number of > 1 min episodes with a MAP < 65 mmHg, n	1 (0, 3)	2 (0, 6)

Data are presented as median (25th percentile, 75th percentile)

MAP, mean arterial pressure

In univariable analysis, age, weight, body mass index, ASA physical status class, arterial hypertension, pre-existing chronic kidney injury, total duration of surgery, and area under a MAP of 65 mmHg were associated with AKI within the first 3 days after surgery (Table 3).

**Table 3:** Univariable associations between variables and acute kidney injury

Variables	OR	95% CI	*P*-value
Age (yr)	1.04	1.01, 1.06	0.002
Weight (kg)	1.01	1.00, 1.02	0.049
Height (cm)	1.01	0.99, 1.04	0.400
ASA physical status class (reference: I)			
II	1.47	0.29, 26.9	0.700
III	4.01	0.81, 72.5	0.200
IV	26.0	1.80, 750	0.022
Body mass index (kg/m^2^)	1.06	1.01, 1.10	0.009
Sex (male vs. female)	1.55	0.91, 2.67	0.110
Body surface area (m^2^)	2.80	0.98, 7.88	0.052
Arterial hypertension (yes vs. no)	1.80	1.06, 3.15	0.034
Diabetes mellitus (yes vs. no)	1.79	0.92, 3.30	0.073
Chronic obstructive pulmonary disease (yes vs. no)	0.94	0.35, 2.11	0.900
Congestive heart failure (yes vs. no)	0.63	0.03, 3.19	0.700
Coronary artery disease (yes vs. no)	1.40	0.52, 3.21	0.500
Prior stroke/transient ischemic attack (yes vs. no)	1.42	0.33, 4.24	0.600
Chronic kidney injury (yes vs. no)	5.62	2.60, 11.6	< 0.001
Total procedure time (min)	1.00	1.00, 1.00	0.039
Area under a MAP of 65 mmHg (mmHg*min)	1.00	1.00, 1.01	< 0.001
Area under a MAP of 65 mmHg per 10 (mmHg*min)	1.04	1.02, 1.06	< 0.001

OR, odds ratio; ASA, American Society of Anesthesiologists; MAP, mean arterial pressure

**Fig. 2:**
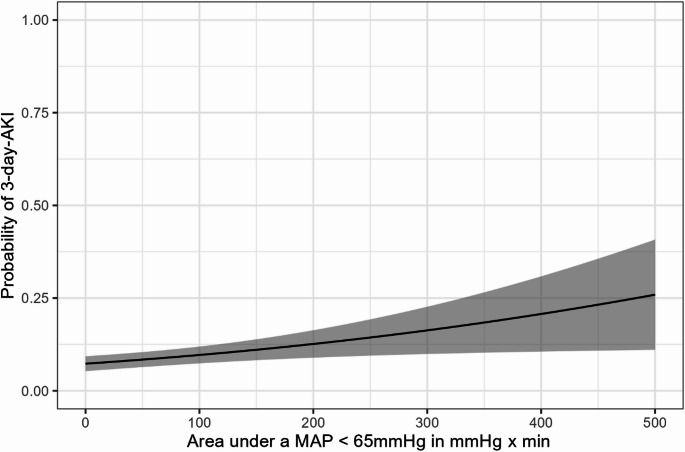
Marginal probability (black line) of developing acute kidney injury (AKI) within the first 3 days after surgery as a function of the area under a mean arterial pressure (MAP) of 65 mmHg (mmHg*min). The probabilities were estimated through g-computation, using the multivariable logistic regression model described in the methods section. Confidence intervals (grey zone) were calculated with the delta method

In multivariable binary logistic regression analysis adjusted for confounding variables, the area under a MAP of 65 mmHg was independently associated with AKI within the first 3 days after surgery (OR 1.03 [95% CI 1.01–1.05] per 10 mmHg*min; *P* < 0.001) (Table 4; Fig. 2).

**Table 4:** Relationship between area under threshold and acute kidney injury: multivariable analysis, adjusted for variables age, body mass index, ASA physical status class, arterial hypertension, pre-existing chronic kidney injury, diabetes mellitus, and total duration of surgery

	Unadjusted			Adjusted		
**Variable**	**OR**	**95% CI**	**P-value**	**OR**	**95% CI**	**P-value**
Area under a MAP of 65 mmHg per 10 (mmHg*min)	1.04	1.02, 1.06	< 0.001	1.03	1.01, 1.05	< 0.001

OR, odds ratio; MAP, mean arterial pressure

## Discussion

This secondary analysis of the EU HYPROTECT registry shows that, in a contemporary population of noncardiac surgery patients in whom clinicians strove to avoid profound intraoperative hypotension, intraoperative hypotension is independently associated with AKI within the first 3 days after surgery. In addition to known risk factors such as age, ASA physical status class, and pre-existing chronic kidney injury, intraoperative hypotension was associated with an increased risk for developing AKI.

Postoperative AKI is an important patient-centred outcome because it is associated with postoperative mortality, prolonged hospitalisation, and the development of chronic kidney injury [21, 22, 23, 24, 25]. AKI incidences reported in noncardiac surgery patients vary depending on the type of surgery and patient risk factors– but can be as high as 18% [18].

Here we show that about 1 in 10 patients developed AKI– although we only considered routinely measured serum creatinine values.

The aetiology of postoperative AKI is multifactorial. Baseline patient risk factors and surgery-related factors are strongly associated with AKI [7, 8]. In our cohort, age, ASA physical status class, pre-existing chronic kidney injury, and the duration of surgery were independently associated with AKI. Patient risk factors and the duration of surgery are not modifiable. To reduce rates of postoperative AKI, modifiable risk factors need to be identified and tackled.

Intraoperative hypotension is a potentially modifiable risk factor for AKI [1, 2, 3, 4, 5, 6, 7, 8, 9, 10]. Our analysis confirms previous registry studies showing an association between intraoperative hypotension and postoperative AKI [1, 2, 3, 4, 5, 6, 7, 8, 9, 10]. In contrast to previous studies, clinical management of our patients included avoiding profound hypotension using predictive monitoring, specifically the HPI-software [15, 16]. Using HPI-software monitoring helped reduce intraoperative hypotension in several studies [26, 27, 28, 29, 30]– including the EU HYPROTECT registry [16]. Specifically, the median time-weighted average MAP < 65 mmHg was 0.03 mmHg in EU HYPROTECT registry patients [16]. As clinicians strove to avoid hypotension in registry patients, the hypotension that occurred presumably was difficult to prevent. Although hypotension was generally uncommon, patients who developed AKI had approximately four times the cumulative area under the MAP of 65 mmHg than those who did not. To quantify the duration and severity of hypotension, we considered the area under a MAP of 65 mmHg and the time-weighted average MAP < 65 mmHg. While time-weighted average MAP is normalised to the duration of monitoring, area under MAP thresholds is probably what is more closely related to organ injury– that is, a function of total hypotension severity and duration [31, 32].

We defined intraoperative hypotension as a MAP below 65 mmHg– which appears to be the population harm threshold for AKI [8, 11]. However, intraoperative hypotensive harm thresholds for individual patients may differ from 65 mmHg because preoperative normal blood pressure also varies among individual patients scheduled for noncardiac surgery [33]. Whether individualising intraoperative MAP targets can help reduce the incidence of postoperative complications is a matter of ongoing research [34].

This is a post hoc secondary analysis of a multicentre registry that includes approximately 700 patients [15, 16]. Although we could confirm that intraoperative hypotension is associated with AKI, our study design does not allow us to conclude that this relationship is causal. Ongoing trials randomising patients to different intraoperative blood pressure targets (e.g., NCT04884802 and NCT05416944 [34]) will help better understand whether universally or individually increasing intraoperative blood pressures can help improve patient outcomes after noncardiac surgery.

Our study has further limitations. Per current recommendations [18, 19], we diagnosed AKI based on clinically available serum creatinine values but did not consider urine output, which often is not reliably assessed after surgery. Not considering urine output, we may, nevertheless, have missed some patients with AKI.

Moreover, in the EU-HYPROTECT cohort, predictive monitoring strategies resulted in very low cumulative durations of hypotension, leading to a highly skewed distribution with limited variability– rendering robust subgroup or regression analyses statistically inappropriate. The prespecified exposure metric, the area under a MAP of 65 mmHg, was chosen based on prior evidence to ensure comparability with earlier studies and to avoid data-driven overfitting. Given the post hoc nature and the limited statistical power of registry data, the analysis was deliberately confined to this single, validated parameter. Another limitation of this study is the lack of a standardized treatment protocol across centres, resulting in inconsistent documentation of fluid therapy, vasopressor/inotrope use, and clinician responses to HPI alerts, which precluded analysis of treatment responses and their association with postoperative outcomes.

## Conclusion

Our secondary analysis of the EU HYPROTECT registry shows that, in a contemporary population of noncardiac surgery patients in whom clinicians strove to avoid profound intraoperative hypotension, intraoperative hypotension is independently associated with AKI within the first 3 days after surgery. Anaesthesiologists should thus aim to avoid profound intraoperative hypotension.

## Data Availability

No datasets were generated or analysed during the current study.
